# A Novel Alignment Method for SINS with Large Misalignment Angles Based on EKF2 and AFIS

**DOI:** 10.3390/s20215975

**Published:** 2020-10-22

**Authors:** Yanming Zhao, Gongmin Yan, Yongyuan Qin, Qiangwen Fu

**Affiliations:** School of Automation, Northwestern Polytechnical University, Xi’an 710129, China; zhaoyanming@mail.nwpu.edu.cn (Y.Z.); qinyongyuan@nwpu.edu.cn (Y.Q.); fuqiangwen@nwpu.edu.cn (Q.F.)

**Keywords:** large misalignment angle, fine alignment, second-order extended Kalman filter (EKF2), adaptive fuzzy inference system (AFIS)

## Abstract

In order to achieve the fine alignment of strapdown inertial navigation (SINS) under large misalignment angles, a novel filtering alignment method is proposed based on the second-order extended Kalman filter (EKF2) and adaptive fuzzy inference system (AFIS). Firstly, the quaternion is employed to represent the attitude errors of SINS. A second-order nonlinear state equation is made based on the nonlinear velocity error model and attitude error model, and the linear measurement equation is based on the velocity outputs from SINS. Then, the filtering scheme is designed based on EKF2 and AFIS. The error estimation and fine alignment can be achieved by using the proposed filtering scheme. The results of Monte Carlo Simulation show that the errors of pitch, roll and yaw misalignment angles quickly decrease to about 14″, 15″ and 7.62′ respectively in 350 s under the condition of any misalignment angles with pitch error from −40° to 40°, roll error from −40° to 40°, and yaw error from −50° to 50°. Even when the initial misalignment angles are all very large such as (80°, 120°, 170°), the proposed nonlinear alignment method still can converge normally by utilizing the adaptive fuzzy inference system (AFIS) to adjust the covariance matrix **P**_k/k−1_. Finally, the turntable experiment was performed, and the effectiveness and superiority of the proposed method were further verified by compared with other nonlinear methods.

## 1. Introduction

The accuracy of strapdown inertial navigation system (SINS) depends largely on the initial alignment [1]. However, under the conditions of low-accuracy inertial sensors or large environmental disturbance, the accuracy of coarse alignment may be very poor, and the fine alignment based on Kalman filter (KF) are no longer applicable [2]. There are some publications on the subject of inertial navigation system (INS) initial alignment on the swaying base, for example, reference [3] proposed a coarse alignment method of marine strapdown INS based on the trajectory fitting of gravity movement in the inertial space, which could avoid the loss of accuracy caused by rocking disturbances. However, there are still some important special application scenarios, for which only the nonlinear alignment methods with the consideration of large initial misalignment angles are applicable: (1) the time of coarse alignment is too short, even if the inertial sensors are sufficiently accurate, the residual misalignment errors could not meet the requirement of small angles for the usage of linear models. In this case, the fine alignment based on the linear error model is difficult to converge to the ultimate precision. For example, when the missile launch vehicle needs to transfer urgently under the threat of artillery attacks, enough time cannot be obtained for the coarse alignment of SINS on the quasi-stationary base. (2) The working environment requires that the fine alignment without coarse alignment process must be achieved directly under the condition of roughly setting the initial attitude, such as missile-borne SINS. With the development of new theories, such as nonlinear filter, robust filter, adaptive estimation, fuzzy inference, and neural network, some approaches combining these new theories with nonlinear filtering method have been proposed for SINS alignment with large misalignment angles. For SINS initial alignment under large misalignment angles, Some alignment methods based on nonlinear filters were designed to improve the estimation precision and convergence speed, which adopted the multi-resetting Kalman filter [4], extended Kalman filter (EKF) [5], scaled unscented Kalman filter (UKF) [5], UKF [6], particle filter (PF) [7], UKF/KF combined filter [8], transformed unscented quadrature Kalman filter (TUQKF) [9], heuristic Kalman filter [10], respectively. In [11] and [12], the methods of SINS in-motion alignment under large misalignment uncertainty were presented, and the nonlinear filters of EKF, UKF, unscented particle filter (UPF), Gaussian particle filter (GPF), Rao-Blackwellized unscented Kalman filter (RBUKF), Rao-Blackwellized unscented particle filter (RBUPF) and Rao-Blackwellized Gaussian particle filter (RBGPF) were compared and analyzed. Under the condition of large misalignment angles, several novel SINS in-motion initial alignment methods using square root nonlinear filters were proposed to improve the filtering stability, which utilized the square root unscented Kalman filter (SRUKF) [13,14], square root cubature information filter (SR-CIF) [15] and square root simplex unscented quadrature Kalman filter (SR-SUQKF) [16], respectively. The adaptive alignment methods based on the Sage–Husa adaptive filter [17] and simplified Sage–Husa adaptive filter [18] were proposed, which could raise the efficiency and adaptability. Aiming at the dynamic disturbance, adaptive Kalman filter was developed, which could be used for the alignment of marine mooring rotary SINS [19] and airborne Micro-Electro-Mechanical-System (MEMS) SINS [20]. By combining nonlinear filters with adaptive estimation, some approaches based on adaptive nonlinear filters were proposed for SINS fine alignment under the conditions of large initial misalignment angles and unknown noise statistics, of which the adaptive nonlinear filters included the adaptive UKF (AUKF) [21,22,23], adaptive cubature Kalman filter (ACKF) [24,25], adaptive unscented particle filter (AUPF) [26,27], adaptive Lie group filter [28], etc. For SINS initial alignment with the nonlinear error model and disturbance noise uncertainty, the H∞ filter was adopted [29,30,31]; thus, the accuracy and robustness of alignment were improved. For SINS initial alignment under inaccurate system model and non-Gaussian observation noise, various fading nonlinear filters were proposed to improve the filtering robustness and convergence, which included the fading UKF filter [32], robust fading CKF filter [33], robust H-infinity CKF/KF hybrid filter (RHCHF) [34], robust adaptive cubature particle filter [35], robust state-dependent Riccati equation (SDRE) filter [36], etc. Combined with fuzzy inference system (FIS), the initial alignment schemes based on fuzzy adaptive filters, such as the fuzzy adaptive Kalman filter [37], fuzzy simplified UKF [38] and fuzzy strong tracking UKF [39,40], etc., were proposed to improve the performance of SINS initial alignment under large alignment angles and disturbance. In [41,42,43,44,45], the SINS initial alignment methods utilizing neural networks were researched, which could be applied for the application scenarios with disturbances, for example, Shipborne, guided projectiles, and so on. In [46,47], two machine learning methods were proposed to achieve the nonlinear initial alignment of SINS under the condition of large misalignment angles, of which one was based on Gaussian process regression (GPR) [46], the other utilized a combination of Gaussian mixture model (GMM), expectation–maximization (EM), and UKF filter [47]. In order to reduce the effects of nonlinear errors, the nonlinear error modeling technique based fast orthogonal search (FOS) was introduced, which have been applied to the radar (RAD)/reduced inertial sensor system (RISS) integration [48], fine frequency estimation of time and code division-orthogonal frequency division multiplexing (TC-OFDM) receivers [49], INS/global navigation satellite system (GNSS) integrated navigation systems [50] and MEMS inertial sensors in mobile devices [51]. However, almost all of these nonlinear alignment methods mentioned above have some shortcomings such as complex algorithm, heavy computational load, difficulties in parameter optimization, insufficient stability, and poor accuracy, etc.

In this paper, a novel initial alignment algorithm based on the second-order extended Kalman filter (EKF2) via adaptive fuzzy inference system (AFIS) is proposed to solve the problem of SINS initial alignment with large misalignment angles. By utilizing the quaternion representation of attitude, the nonlinear error equations of SINS are derived, and the state equation is obtained. On this basis, the velocity information of SINS is used to establish the measurement equation of the nonlinear alignment with large misalignment angles. The adaptive fuzzy inference system and parameter adaptive estimation are used to assist the second-order EKF filter, also referred to as the second-order EKF filter assisted by adaptive fuzzy inference system (‘AFIS-EKF2’ or ‘EKF2 via AFIS’ for short), and the algorithm of EKF2 via AFIS is designed. The filtering process of EKF2 via AFIS is performed to obtain the estimation of misalignment angles, and the fine alignment of SINS with large misalignment angles is achieved. As a result, the proposed large misalignment angle alignment algorithm is effective in achieving the SINS fine alignment with large alignment angles.

The rest of this paper is organized as follows: Section 2 derives the nonlinear error equations of SINS with large misalignment angles by using the quaternion representation of attitude. In Section 3, the filtering model of SINS nonlinear alignment is established by using the velocity information of SINS as the measurement of nonlinear estimation, and the algorithm of simplified second-order EKF filter is designed. Section 4 presents the strong tracking strategy and fuzzy adaptive parameter adjustment method. In Section 5, Experiment setup and result analysis are provided, which include the simulations of fine alignment on stationary base/swaying base and the experiment on three-axis turntable. In addition, the content of this paper is summarized in Section 6.

## 2. Nonlinear Error Equations of SINS with Large Misalignment Angles

The reference frames in use are denoted as: the script i denotes the Earth-Centered Inertial (ECI) frame, n denotes the navigation frame (East–North–Up, ENU), e denotes the Earth-Centered Earth-Fixed frame (ECEF), and b denotes the body-fixed frame. In order to distinguish the error-free variable from the corresponding variable with error, different variants of the same script are used to represent the true value, calculated value and measured value, respectively. For example, the script x denotes the error-free true value of any variable, then x hat ($\hat{x}$) and x tilde ($\tilde{x}$) denote the corresponding calculated value and measured value, respectively.

### 2.1. Attitude Error Equation

When no error is considered, the differential equation of attitude quaternion is expressed as

(1) $$\dot{Q}=\frac{1}{2}Q⊗\omega_{nb}^{b}$$

When considering various error resources, the differential equation of attitude quaternion is expressed as

(2) $$\dot{\hat{Q}}=\frac{1}{2}\hat{Q}⊗{\hat{\omega }}_{nb}^{b}$$

where Q represents the true value of attitude quaternion, $\hat{Q}$ represents the calculated value of SINS attitude quaternion, ⊗ represents the multiplication of quaternions, $\omega_{nb}^{b}$ represents the angular velocity of the *b*-frame relative to the *n*-frame expressed in the *b*-frame, ${\hat{\omega }}_{nb}^{b}$ represents the calculated value of $\omega_{nb}^{b}$.

The calculating method of $\omega_{nb}^{b}$ and ${\hat{\omega }}_{nb}^{b}$ is as follows:

(3) $$\omega_{nb}^{b}=\omega_{ib}^{b}-Q^{*}⊗\omega_{in}^{n}⊗Q$$

(4) $${\hat{\omega }}_{nb}^{b}={\tilde{\omega }}_{ib}^{b}-{\hat{Q}}^{*}⊗{\hat{\omega }}_{in}^{n}⊗\hat{Q}=\left(\omega_{ib}^{b}+\delta \omega_{ib}^{b}\right)-{\hat{Q}}^{*}⊗\left(\omega_{in}^{n}+\delta \omega_{in}^{n}\right)⊗\hat{Q}$$

where $Q^{*}$ and ${\hat{Q}}^{*}$ represent the conjugates of Q and $\hat{Q}$, respectively. $\omega_{ib}^{b}$ is the angular velocity of the b-frame relative to the i-frame expressed in the b-frame, $\omega_{in}^{n}$ is the angular velocity of the n-frame relative to the i-frame expressed in the n-frame. ${\tilde{\omega }}_{ib}^{b}$ is the measured value of $\omega_{ib}^{b}$, which is obtained from the gyro outputs. ${\hat{\omega }}_{in}^{n}$ is the calculated value of $\omega_{in}^{n}$. $\delta \omega_{ib}^{b}$ represents the measurement error of $\omega_{ib}^{b}$, which is mainly gyro drift. $\delta \omega_{in}^{n}$ represents the calculation error of $\omega_{in}^{n}$, which is mainly related to the velocity error and position error of the vehicle.

Define the error quaternion δQ as

(5) $$\delta Q=Q⊗{\hat{Q}}^{*}$$

where ${\hat{Q}}^{*}$ is the conjugate of calculated quaternion $\hat{Q}$.

According to Equation (5), the derivative of δQ with respective to t can be expressed as

(6) $$\delta \dot{Q}=\dot{Q}⊗{\hat{Q}}^{*}+Q⊗{\dot{\hat{Q}}}^{*}$$

The differential equation of δQ can be obtained by substituting Equations (1) and (2) into Equation (6), as shown in Equation (7):

(7) $$\begin{array}{cc} \delta \dot{Q} & =-\frac{1}{2}Q⊗\left({\tilde{\omega }}_{ib}^{b}-\omega_{ib}^{b}\right)⊗{\hat{Q}}^{*}-\frac{1}{2}Q⊗\omega_{in}^{b}⊗{\hat{Q}}^{*}+\frac{1}{2}Q⊗{\hat{\omega }}_{in}^{b}⊗{\hat{Q}}^{*}\\ & =-\frac{1}{2}Q⊗\delta {\tilde{\omega }}_{ib}^{b}⊗{\hat{Q}}^{*}-\frac{1}{2}Q⊗\omega_{in}^{b}⊗{\hat{Q}}^{*}+\frac{1}{2}Q⊗{\hat{\omega }}_{in}^{b}⊗{\hat{Q}}^{*}\\ & =-\frac{1}{2}Q⊗{\hat{Q}}^{*}⊗\hat{Q}⊗\delta {\tilde{\omega }}_{ib}^{b}⊗{\hat{Q}}^{*}-\frac{1}{2}Q⊗Q^{*}⊗({\hat{\omega }}_{in}^{n}-\delta \omega_{in}^{n})⊗Q⊗{\hat{Q}}^{*}+\frac{1}{2}Q⊗{\hat{Q}}^{*}⊗{\hat{\omega }}_{in}^{n}⊗\hat{Q}⊗{\hat{Q}}^{*}\\ & =-\frac{1}{2}\delta Q⊗\delta {\tilde{\omega }}_{ib}^{n}-\frac{1}{2}({\hat{\omega }}_{in}^{n}-\delta \omega_{in}^{n})⊗\delta Q+\frac{1}{2}\delta Q⊗{\hat{\omega }}_{in}^{n} \end{array}$$

where

$$\left\{\begin{array}{l} {\hat{\omega }}_{in}^{n}=\omega_{in}^{n}+\delta \omega_{in}^{n}\\ {\hat{\omega }}_{in}^{b}={\hat{Q}}^{*}⊗{\hat{\omega }}_{in}^{n}⊗\hat{Q}\\ \omega_{in}^{b}=Q^{*}⊗\omega_{in}^{n}⊗Q\\ \delta {\tilde{\omega }}_{ib}^{b}={\tilde{\omega }}_{ib}^{b}-\omega_{ib}^{b}\\ \delta {\tilde{\omega }}_{ib}^{n}=\hat{Q}⊗\delta {\tilde{\omega }}_{ib}^{b}⊗{\hat{Q}}^{*} \end{array}\right.$$

Equation (7) is the attitude error equation of SINS under the condition of large misalignment angles.

During the fine alignment on quasi-stationary base, the position information of vehicle can be accurately obtained, and the velocity of vehicle can be approximated as 0 m/s. By utilizing the information of zero-velocity and only considering the correlative bias of inertial sensor error, the attitude error equation is simplified as follows:

(8) $$\begin{array}{ll} \delta \dot{Q} & =-\frac{1}{2}\delta Q⊗\delta {\tilde{\omega }}_{ib}^{n}-\frac{1}{2}{\hat{\omega }}_{ie}^{n}⊗\delta Q+\frac{1}{2}\delta Q⊗{\hat{\omega }}_{ie}^{n}\\ & =-\frac{1}{2}\delta Q⊗\varepsilon_{b}^{n}-\frac{1}{2}{\hat{\omega }}_{ie}^{n}⊗\delta Q+\frac{1}{2}\delta Q⊗{\hat{\omega }}_{ie}^{n}-\frac{1}{2}\delta Q⊗\varepsilon_{w}^{n} \end{array}$$

where $\varepsilon_{b}^{n}$ and $\varepsilon_{w}^{n}$ are the random constant bias and white noise of the gyro, respectively; ${\hat{\omega }}_{ie}^{n}$ is the calculated angular velocity of earth rotation in the n-frame.

### 2.2. Velocity Error Equation

Without considering any errors, the velocity equation of SINS is expressed as follows:

(9) $${\dot{V}}^{n}=C_{b}^{n}f_{sf}^{b}-\left(2\omega_{ie}^{n}+\omega_{en}^{n}\right)\times V^{n}+g_{}^{n}$$

Considering various error resources, the velocity equation of SINS is:

(10) $${\dot{\hat{V}}}^{n}={\hat{C}}_{b}^{n}{\tilde{f}}_{sf}^{b}-\left(2{\hat{\omega }}_{ie}^{n}+{\hat{\omega }}_{en}^{n}\right)\times {\hat{V}}^{n}+{\hat{g}}_{}^{n}$$

where $V^{n}$ represents the true vehicle velocity, ${\hat{V}}^{n}$ represents the calculated vehicle velocity of SINS, $\delta V^{n}$ represents the velocity error of SINS, and ${\hat{V}}^{n}=V^{n}+\delta V^{n}$; $C_{b}^{n}$ represents the true attitude matrix, ${\hat{C}}_{b}^{n}$ represents the calculated attitude matrix of SINS, $\delta C_{n}^{n^{\prime }}$ represents the attitude error matrix of SINS, and ${\hat{C}}_{b}^{n}=\delta C_{n}^{n^{\prime }}C_{b}^{n}$; $f_{sf}^{b}$ is the true specific force on the vehicle, ${\tilde{f}}_{sf}^{b}$ represents the measured specific force, which is obtained by the output of accelerators. $\delta f_{sf}^{b}$ represents the measurement error of $f_{sf}^{b}$, which is mainly accelerator bias, and ${\tilde{f}}_{sf}^{b}=f_{sf}^{b}+\delta f_{sf}^{b}$; $\omega_{ie}^{n}$ represents the angular velocity of earth rotation in the n-frame, ${\hat{\omega }}_{ie}^{n}$ represents the calculated value of $\omega_{ie}^{n}$, which is related to the position of SINS, $\delta \omega_{ie}^{n}$ represents the calculation error of ${\hat{\omega }}_{ie}^{n}$, which is mainly caused by the position error of SINS, and ${\hat{\omega }}_{ie}^{n}=\omega_{ie}^{n}+\delta \omega_{ie}^{n}$; $\omega_{en}^{n}$ represents the angular velocity of the n-frame relative to the e-frame coordinated in the n-frame, ${\hat{\omega }}_{en}^{n}$ represents the calculated value of $\omega_{en}^{n}$; $\delta \omega_{en}^{n}$ represents the calculation error of ${\hat{\omega }}_{en}^{n}$, and ${\hat{\omega }}_{en}^{n}=\omega_{en}^{n}+\delta \omega_{en}^{n}$; $g_{}^{n}$ represents the gravity acceleration expressed in the n-frame, ${\hat{g}}_{}^{n}$ represents the calculated value of $g_{}^{n}$, $\delta g_{}^{n}$ represents the calculation error of ${\hat{g}}_{}^{n}$, and ${\hat{g}}_{}^{n}=g_{}^{n}+\delta g_{}^{n}$.

By subtracting Equation (9) from Equation (10), and rearranging the error terms, the differential equation of SINS velocity error under large misalignment angles can be obtained, as shown in Equation (11).

(11) $$\delta {\dot{V}}^{n}=\left(I_{3\times 3}^{}-C_{n^{\prime }}^{n}\right){\hat{C}}_{b}^{n}{\tilde{f}}_{sf}^{b}-\left(2{\hat{\omega }}_{ie}^{n}+{\hat{\omega }}_{en}^{n}\right)\times \delta V^{n}-\left(2\delta \omega_{ie}^{n}+\delta \omega_{en}^{n}\right)\times {\hat{V}}^{n}+C_{n^{\prime }}^{n}{\hat{C}}_{b}^{n}\nabla_{b}^{b}+C_{n^{\prime }}^{n}{\hat{C}}_{b}^{n}\nabla_{w}^{b}$$

where $\nabla_{b}^{b}$ and $\nabla_{w}^{b}$ are the random constant bias and white noise of the accelerometer, respectively.

Similarly, under the condition of zero-velocity, the velocity error equation is simplified as:

(12) $$\delta {\dot{V}}^{n}\approx \left(I_{3\times 3}^{}-C_{n^{\prime }}^{n}\right){\hat{C}}_{b}^{n}{\tilde{f}}_{sf}^{b}+C_{n^{\prime }}^{n}{\hat{C}}_{b}^{n}\nabla_{b}^{b}+C_{n^{\prime }}^{n}{\hat{C}}_{b}^{n}\nabla_{w}^{b}$$

## 3. Nonlinear Filtering Model and Second-Order EKF Algorithm

### 3.1. Filtering Model of SINS Nonlinear Alignment with Large Misalignment Angles

For the errors of inertial sensors, only the random constant bias and white noise are considered. The gyro constant drift and accelerometer constant bias are augmented into the system state vector, and the system state vector is defined as

(13) $$X\left(t\right)={\left[\begin{array}{ccc} \delta Q^{T} & \delta V^{nT} & \begin{array}{cc} \varepsilon_{b}^{bT} & \nabla_{b}^{bT} \end{array} \end{array}\right]}^{T}$$

where $\delta Q={\left[\begin{array}{cccc} \delta q_{0} & \delta q_{1} & \delta q_{2} & \delta q_{3} \end{array}\right]}_{}^{T}$ is the error quaternion, $\delta V^{n}={\left[\begin{array}{ccc} \delta V_{E}^{n} & \delta V_{N}^{n} & \delta V_{U}^{n} \end{array}\right]}^{T}$ is the velocity error, $\varepsilon_{b}^{b}={\left[\begin{array}{ccc} \varepsilon_{b,x}^{b} & \varepsilon_{b,y}^{b} & \varepsilon_{b,z}^{b} \end{array}\right]}^{T}$ is the gyro random constant drift, and $\nabla_{b}^{b}={\left[\begin{array}{ccc} \nabla_{b,x}^{b} & \nabla_{b,y}^{b} & \nabla_{b,z}^{b} \end{array}\right]}^{T}$ is the accelerometer random constant bias.

According to the nonlinear error equations of SINS, the system state equation for nonlinear filter can be established as follows:

(14) $$\dot{X}\left(t\right)=f[X\left(t\right),t]+G\left(t\right)w\left(t\right)$$

(15) $$f[X\left(t\right),t]=\left[\begin{array}{c} -\frac{1}{2}\delta Q⊗\hat{Q}⊗\varepsilon_{b}^{b}⊗{\hat{Q}}^{*}-\frac{1}{2}{\hat{\omega }}_{ie}^{n}⊗\delta Q+\frac{1}{2}\delta Q⊗{\hat{\omega }}_{ie}^{n}\\ \left(I_{3\times 3}^{}-C_{n^{\prime }}^{n}\right){\hat{C}}_{b}^{n}{\tilde{f}}_{sf}^{b}+C_{n^{\prime }}^{n}{\hat{C}}_{b}^{n}\nabla_{b}^{b}\\ 0_{3\times 1}\\ 0_{3\times 1} \end{array}\right]$$

where $G\left(t\right)$ represents the system noise driving matrix, $w\left(t\right)={\left[\begin{array}{ccc} 0 & \varepsilon_{w}^{bT} & \nabla_{w}^{bT} \end{array}\right]}_{}^{T}$ represents the system driving noise, $\varepsilon_{w}^{bT}$ and $\nabla_{w}^{bT}$ represent the white noises of gyroscopes and accelerometers, respectively. The expression of system noise driving matrix $G\left(t\right)$ is shown in Equation (16):

(16) $$G\left(t\right)=\left[\begin{array}{cc} -\frac{1}{2}{\left[\hat{Q}\right]}_{L}{\left[{\hat{Q}}^{*}\right]}_{R} & 0_{4\times 3}\\ 0_{3\times 4}^{} & {\hat{C}}_{b}^{n}\\ 0_{6\times 4}^{} & 0_{6\times 3}^{} \end{array}\right]$$

where ${\left[\times \right]}_{L}$ and ${\left[\times \right]}_{R}$ are two different 4×4 matrices, which are both composed of the elements of corresponding quaternion ×. Moreover, they are convenient to realize the quaternion multiplication by using matrix multiplication. The matrices ${\left[\times \right]}_{L}$ and ${\left[\times \right]}_{R}$ are defined as follows:

(17) $$P⊗Q≜{\left[P\right]}_{L}Q≜{\left[Q\right]}_{R}P$$

The velocity of SINS is taken as the measurement of nonlinear filtering alignment, the measurement equation of SINS nonlinear alignment can be constructed as

(18) $$Z_{k}=\delta V_{k}^{n}=H_{k}^{}X_{k}^{}+V_{k}$$

where $H_{k}^{}=\left[\begin{array}{ccc} 0_{3\times 4} & I_{3\times 3} & \begin{array}{cc} 0_{3\times 3} & 0_{3\times 3} \end{array} \end{array}\right]$ and $V_{k}$ is the measurement noise which corresponds to the measurement error resulting from the environmental disturbance.

According to Equations (14) and (18), the nonlinear filtering model in state space is obtained, and can be presented as

(19) $$\left\{\begin{array}{l} \dot{X}\left(t\right)=f[X\left(t\right),t]+G\left(t\right)w\left(t\right)\\ Z_{k}=H_{k}^{}X_{k}^{}+V_{k} \end{array}\right.$$

### 3.2. Simplified Second-Order EKF Algorithm

As shown in Equation (20), the nonlinear filtering model obtained in the previous subsection is copied into this subsection, which includes the system state equation and measurement equation.

(20) $$\left\{\begin{array}{l} \dot{X}\left(t\right)=f[X\left(t\right),t]+G\left(t\right)w\left(t\right)\\ Z_{k}=H_{k}^{}X_{k}^{}+V_{k} \end{array}\right.$$

where $X\left(t\right)$ represents the system state vector at time t; $f[X\left(t\right),t]$ represents the nonlinear function of $X\left(t\right)$ which describes the system state equation with continuous form; the subscript k represents the time $t_{k}^{}$; $Z_{k}$ represents the measurement vector; $H_{k}^{}$ represents the measurement matrix; $G\left(t\right)$ represents the system noise driving matrix; $w\left(t\right)$ represents the continuous system noise, which is assumed as a zero-mean Gaussian white noise with covariance $q\left(t\right)$; $V_{k}$ represents the discrete measurement noise sequence, which is assumed as a zero-mean Gaussian white noise sequence with covariance $R_{k}$; and assuming that $w\left(t\right)$ is uncorrelated with $V_{k}$; the dimensions of $X\left(t\right)$ and $Z_{k}$ are denoted as n and m; in this paper, n=13 and m=3.

It can be seen from Equation (20) that the system state equation is nonlinear, and the measurement equation is linear. The second-order EKF algorithm can be simplified, and this simplified algorithm is abbreviated as SEKF2. By using the fourth-order Runge–Kutta method, the time update process of SEKF2 is implemented based on the second-order Taylor expansion of nonlinear vector function in state equation. The measurement update process of SEKF2 is the same as that of classical linear Kalman filter. The steps of SEKF2 are as follows:

(1) Selection of Initial Filtering Parameters

(21) $${\hat{X}}_{0}^{}=E\left[X_{0}^{}\right],P_{0}^{}=E\left[\left({\hat{X}}_{0}^{}-X_{0}^{}\right){\left({\hat{X}}_{0}^{}-X_{0}^{}\right)}_{}^{T}\right]$$

(2) Time Update

(22) $${\dot{\hat{X}}}^{-}\left(t\right)=f[{\hat{X}}^{-}\left(t\right),t]+\frac{1}{2}\sum_{i=1}^{n}e_{i}Tr\left[{\left.\left(\nabla \nabla^{T}f_{i}\right)\right|}_{X\left(t\right)={\hat{X}}^{-}\left(t\right)}P^{-}\left(t\right)\right]$$

(23) $${\dot{P}}^{-}\left(t\right)=F\left(t\right)P^{-}\left(t\right)+P^{-}\left(t\right)F^{T}\left(t\right)+G\left(t\right)qG^{T}\left(t\right)$$

where $f_{i}$ is the i-th component of the nonlinear vector function $f[X\left(t\right),t]$; $e_{i}$ is the unit vector with the i-th component being 1 and the remaining components being 0; $F\left(t\right)$ is the Jacobian matrix of $f[X\left(t\right),t]$ with respect to $X\left(t\right)$; $\nabla \nabla^{T}f_{i}$ is the Hessian matrix of $f_{i}$ with respect to $X\left(t\right)$; The expressions of $F\left(t\right)$ and $\nabla \nabla^{T}f_{i}$ are shown in Equation (24):

(24) $$\left\{\begin{array}{l} F\left(t\right)={\left.\left(\partial f/\partial X^{T}\left(t\right)\right)\right|}_{X\left(t\right)={\hat{X}}^{-}\left(t\right)}\\ \nabla \nabla^{T}f_{i}={\left.\left(\partial \left({\left(\partial f_{i}/\partial X^{T}\left(t\right)\right)}^{T}\right)/\partial X^{T}\left(t\right)\right)\right|}_{X\left(t\right)={\hat{X}}^{-}\left(t\right)} \end{array}\right.$$

By using the fourth-order Runge-Kutta method, the differential Equations (22) and (23) are integrated on the filtering period $\left[t_{k-1},t_{k}\right]$. Thus the one-step predictive estimations of state X and covariance P can be obtained, which are denoted as ${\hat{X}}_{k/k-1},\;P_{k/k-1}$, respectively. In the integral process, the initial values are ${\hat{X}}^{-}\left(t_{k-1}\right)={\hat{X}}_{k-1}$ and $P^{-}\left(t_{k-1}\right)=P_{k-1}$, and the final values ${\hat{X}}_{k/k-1}={\hat{X}}^{-}\left(t_{k}\right)$ and $P_{k/k-1}=P^{-}\left(t_{k}\right)$ will be used in the measurement update process.

(3) Measurement Update

(25) $${\hat{Z}}_{k/k-1}^{}=H_{k}^{}{\hat{X}}_{k/k-1}^{}$$

(26) $$P_{XZ,k/k-1}^{}=P_{k/k-1}^{}H_{k}^{T}$$

(27) $$P_{ZZ,k/k-1}^{}=H_{k}^{}P_{k/k-1}^{}H_{k}^{T}+R_{k}^{}$$

(28) $$K_{k}^{}=P_{XZ,k/k-1}^{}P_{ZZ,k/k-1}^{-1}$$

(29) $${\hat{X}}_{k}^{}={\hat{X}}_{k/k-1}^{}+K_{k}^{}\left(Z_{k}^{}-{\hat{Z}}_{k/k-1}^{}\right)$$

(30) $$P_{k}^{}=P_{k/k-1}^{}-K_{k}^{}P_{ZZ,k/k-1}^{}K_{k}^{T}$$

where ${\hat{Z}}_{k/k-1}^{}$ is the one-step prediction of measurement $Z_{k}$, $P_{XZ,k/k-1}^{}$ is the cross-covariance matrix of ${\hat{Z}}_{k/k-1}^{}$ and ${\hat{X}}_{k/k-1}^{}$, $P_{ZZ,k/k-1}^{}$ is the auto-covariance matrix of ${\hat{Z}}_{k/k-1}^{}$, $R_{k}^{}$ is the covariance matrix of measurement noise sequence $V_{k}$, $K_{k}^{}$ is the gain matrix, ${\hat{X}}_{k}^{}$ and $P_{k}^{}$ are the optimal estimation of system state and the covariance matrix of state estimation error at time $t_{k}^{}$, respectively.

## 4. The Algorithms of Strong Tracking Strategy and Fuzzy Adaptive Parameter Adjustment

### 4.1. Strong Tracking Strategy

Under the condition that the filtering model and noise statistics are accurate, the optimal estimation of state can be obtained by using KF. However, under the conditions of inaccurate filtering model and noise statistics, the estimation error of KF will be increased or even divergent. To solve the problem of filtering divergence caused by inaccurate state equation or non-Gaussian system noise, strong tracking Kalman filter (STKF) was proposed, which is based on the orthogonality of innovation sequence [52,53,54].

In KF filtering, the innovation $e_{k}$ is defined as

(31) $$e_{k}=Z_{k}-H_{k}{\hat{X}}_{k/k-1}$$

Under the assumption of white Gaussian noise, the expectation of innovation covariance is expressed by Equation (32):

(32) $$E\left(e_{k}e_{k}^{T}\right)=E_{k}^{}≜H_{k}^{}P_{k/k-1}^{}H_{k}^{T}+R_{k}^{}$$

When the process noise is non-Gaussian or the system model is inaccurate, the differences between the calculated value ${\hat{E}}_{k}^{}$ and theoretical value $E_{k}^{}$ of innovation covariance would be significantly large. In this case, the confidence degree of state prediction should be decreased, and the confidence degree of current measurement value should be increased. For the purpose of adjusting the confidence degrees, the value of $P_{k/k-1}$ is increased by introducing attenuation factor $\gamma_{k}^{}$, and the attenuation factor $\gamma_{k}^{}$ is calculated according to the orthogonality of innovation sequence.

According to the filtering model, the covariance matrix of innovations at two different time instants is expressed as

(33) $$E\left(e_{k+j}e_{k}^{T}\right)=H_{k+j}^{}\cdot \prod_{l=k+1}^{k+j-1}\Phi_{l/l-1}^{}\left(I-K_{l}^{}H_{l}^{}\right)\cdot \Phi_{k/k-1}^{}\left(P_{k/k-1}^{}H_{k}^{T}-K_{k}^{}E_{k}^{}\right)j=1,2,\ldots$$

It can be seen that a sufficient condition for the orthogonality of innovation sequence is

(34) $$S_{k}^{}=P_{k/k-1}^{}H_{k}^{T}-K_{k}^{}E_{k}^{}=0$$

By substituting the filter gain matrix Equation (28) into (34), the sufficient condition could be rewritten as

(35) $$P_{k/k-1}^{}H_{k}^{T}-P_{k/k-1}^{}H_{k}^{T}{\left(H_{k}^{}P_{k/k-1}^{}H_{k}^{T}+R_{k}^{}\right)}_{}^{-1}E_{k}^{}=0$$

Under the condition of measurement matrix $H_{k}^{}$ with full column rank, the equivalent form is obtained, as shown in Equation (36):

(36) $$E_{k}^{}=H_{k}^{}P_{k/k-1}^{}H_{k}^{T}+R_{k}^{}$$

By comparing Equations (32) and (36), it can be readily known that, under the assumptions of the accurate filtering model and Gaussian process noise, the innovation of Kalman filter meets the orthogonality. However, if the above assumptions are not satisfied, the difference between ${\hat{E}}_{k}^{}$ and $E_{k}^{}$ will be relatively large, and the innovation will not meet the orthogonality. In order to keep orthogonality of innovation sequence, $P_{k/k-1}$ should be modified by utilizing $\gamma_{k}^{}$, as shown in Equation (37):

(37) $$P_{k/k-1}=\gamma_{k}^{}P_{k/k-1}$$

Substituting Equation (37) into (36), $E_{k}^{}$ is modified as

(38) $${\hat{E}}_{k}^{}=\gamma_{k}^{}H_{k}^{}P_{k/k-1}^{}H_{k}^{T}+\eta R_{k}^{}$$

where η represents the softening factor, the value of $R_{k}^{}$ can be adjusted by presetting η, thus the confidence degree of measurement $Z_{k}^{}$ can be directly adjusted.

Based on Equation (38), the attenuation factor $\gamma_{k}^{}$ can be obtained by using the operations of calculating the matrix trace and absolute value, as shown in Equation (39).

(39) $$\gamma_{k}^{}=0.99\cdot \left|\mathrm{tr}\left({\hat{E}}_{k}^{}-\eta R_{k}^{}\right)/\mathrm{tr}\left(H_{k}^{}P_{k/k-1}^{}H_{k}^{T}\right)\right|$$

where the coefficient 0.99 is a preset constant, which is adjusted according to the actual conditions.

In order to better modify $P_{k/k-1}^{}$, the attenuation factor $\gamma_{k}^{}$ is selected as

(40) $$\gamma_{k}^{}\in \left\{1,0.99\cdot \left|\mathrm{tr}\left({\hat{E}}_{k}^{}-\eta R_{k}^{}\right)/\mathrm{tr}\left(H_{k}^{}P_{k/k-1}^{}H_{k}^{T}\right)\right|\right\}$$

where the calculated value of innovation covariance matrix ${\hat{E}}_{k}^{}$ is selected as follows:

(41) $${\hat{E}}_{k}=\left\{\begin{array}{l} e_{k}e_{k}^{T},\;k=1\\ \left(\rho {\hat{E}}_{k-1}+e_{k}e_{k}^{T}\right)/\left(1+\rho \right),\;k>1 \end{array}\right.$$

where $\rho \in \left(0,1\right)$ represents the forgetting factor, of which the value is usually 0.95.

From the above analysis, it can be concluded that the non-orthogonality of innovation sequence is caused by the non-Gaussian process noise or inaccurate system model. When the process noise or state model is abnormal, the attenuation factor $\gamma_{k}^{}$ of STKF is adaptively adjusted. Moreover, the prediction estimation variance matrix (PECM) $P_{k/k-1}^{}$ of STKF is enlarged to increase the weight of current innovation (or measurement) in filtering estimation. By decreasing the confidence of one-step prediction, the filter gain matrix $K_{k}$ is adjusted in real-time, thus the state estimation can be satisfied for the requirements of minimum variance and the innovation sequence is forced to keep orthogonal to each other. As a result, the effective information in the innovation sequence is extracted to the maximum extent. Under the conditions of inaccurate system model and non-Gaussian process noise, STKF has the ability to track the system state, robustness on model mismatch and system disturbance, and high accuracy and stability. Combined with strong tracking strategy, EKF2 can be modified to deal with the nonlinear filtering alignment with model uncertainties.

### 4.2. Fuzzy Adaptive Parameter Adjustment

**Figure 1:**
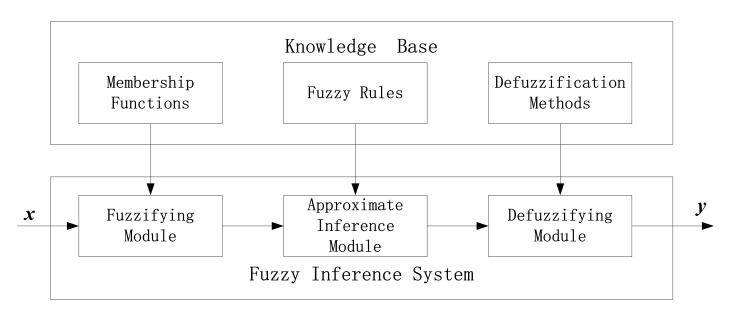
Schematic diagram of a standard fuzzy logic system.

In 1965, Professor L.A. Zadeh proposed the theory of fuzzy sets and fuzzy logic at the University of California, which contributed to the progress of reasoning with imprecise concepts and provided a new approach to qualitatively represent human knowledge. The principle of fuzzy inference system is shown in Figure 1. By utilizing the fuzzy inference, the mapping from input space to output space is realized in the fuzzy logic system (FLS). Fuzzy rules are the core of fuzzy inference system, and they are generally expressed by the “if-then” rule statements.

**Figure 2:**
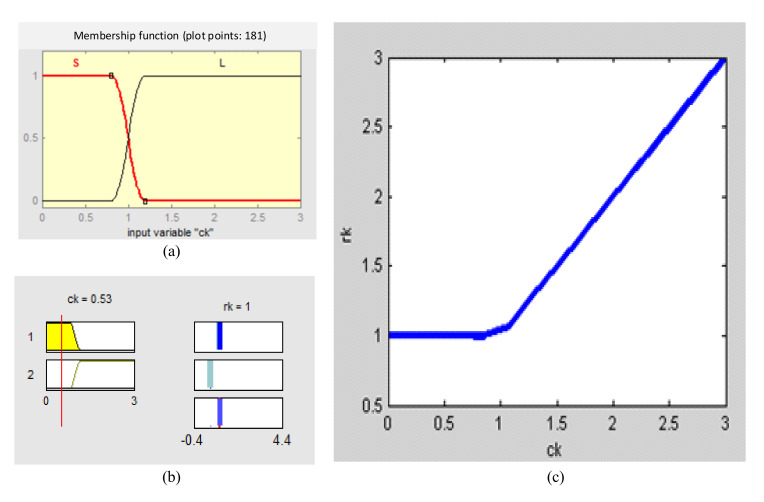
The membership function curves/rule viewer/input–output surface viewer. (**a**) The membership function; (**b**) the rule viewer; (**c**) the input–output surface viewer.

For the convenience in designing a fuzzy inference system(FIS) to realize the strong tracking strategy better, the part of $0.99\left|tr\left({\hat{E}}_{k}-\eta R_{k}\right)/tr\left(H_{k}^{}P_{k/k-1}H_{k}^{T}\right)\right|$ in Equation (40) is denoted as $c_{k}^{}$, i.e., $c_{k}^{}≜0.99\left|tr\left({\hat{E}}_{k}-\eta R_{k}\right)/tr\left(H_{k}^{}P_{k/k-1}H_{k}^{T}\right)\right|$. Then the fuzzy inference system with the form of single- input single-output(SISO) is designed, in which the parameters $c_{k}^{}$ and $\gamma_{k}^{}$ are selected as the input and output, respectively. From the strong tracking theory and engineering experience, it can be obtained that: if the value of $c_{k}^{}$ is relatively large, it means that the difference between ${\hat{E}}_{k}$ and $E_{k}$ is relatively large, that is to say the errors of system model or prior information are relatively large, then the strong tracking function is enabled, i.e., letting $\gamma_{k}^{}=c_{k}^{}$; Conversely, if the value of $c_{k}^{}$ is relatively small, it means that the difference between ${\hat{E}}_{k}$ and $E_{k}$ is relatively small, that is to say the system model and prior information are accurate, then the strong tracking function is disabled and only the EKF2 filter is carried out, i.e., letting $\gamma_{k}^{}=1$. Two fuzzy subsets are defined to cover the input variable domain, and they are referred to as Small (S) and Large (L). The Z-shaped curve membership function (zmf) is selected for the fuzzy subset S and S-shaped curve membership function (smf) is selected for the fuzzy subset L. The fuzzy subsets and membership functions of the input variable $c_{k}^{}$ are shown in Table 1. The fuzzy inference system is designed, and its membership functions, fuzzy rules and input-output surface are shown in Figure 2a–c. In this paper, Sugeno-type fuzzy inference system is adopted, and two fuzzy rules are established as follows:

$$\begin{array}{l} R_{}^{1}:if\left(c_{k}^{}\mathrm{is}S\right)\;\mathrm{then}\;\left(\gamma_{k}^{}=1\right);\\ R_{}^{2}:if\left(c_{k}^{}\mathrm{is}L\right)\;\mathrm{then}\;\left(\gamma_{k}^{}=c_{k}^{}\right); \end{array}$$

**Figure 3:**
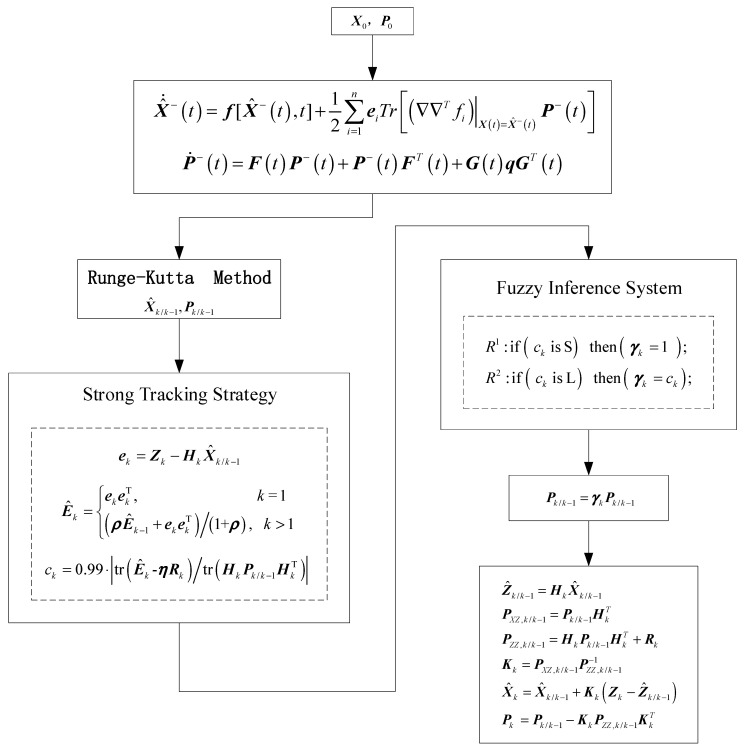
Flowchart of the second-order extended Kalman filter (EKF2) filter assisted by the adaptive fuzzy inference system (AFIS).

By combining the second-order EKF filter and fuzzy adaptive parameter adjustment, the algorithm of the second-order extended Kalman filter (EKF2) filter assisted by the adaptive fuzzy inference system (AFIS) can be obtained, of which the flowchart is shown in Figure 3.

## 5. Experiment Setup and Result Analysis

### 5.1. The Simulation of Fine Alignment on Stationary Base

In order to analyze the precision of the algorithm, several simulative analyses of AFIS-EKF2 filtering alignment were carried out, and the simulation results were analyzed statistically. In the simulations, the initial attitude errors and inertial measurement unit (IMU) errors were preset, and the IMU data on static base were generated by trajectory simulation.

#### 5.1.1. Simulation Conditions

By using MATLAB software (MATLAB R2009a, The MathWorks. Inc.), we performed simulation with the conditions as follows:

(1) The State of Vehicle.

The vehicle is stationary, and the position is: longitude 108.9°E, latitude 34.2°N, altitude 400 m; the velocity of vehicle is 0 m/s; and the attitude of vehicle is 5°, 10°, 45°.

(2) IMU Errors.

Gyro errors: the random constant drift is 0.02°/h, the angular random walk coefficient is $0.003{}^{\circ}/\sqrt{h}$.

Accelerometer errors: the random constant bias is 0.05 mg, the velocity random walk coefficient is $0.01\;\mathrm{mg}/\sqrt{\mathrm{Hz}}$.

(3) Parameter Setting of Filter.

The velocity measurement noise of SINS on stationary base is assumed to be Gaussian white noise with amplitude of $0.05\;m/s\left(1\sigma \right)$; the filtering period is 1 s; the correction mode is selected as feedback correction. The alignment accuracy is evaluated by using the difference between the fine alignment results and the true attitudes.

#### 5.1.2. Simulation Results

**Figure 4:**
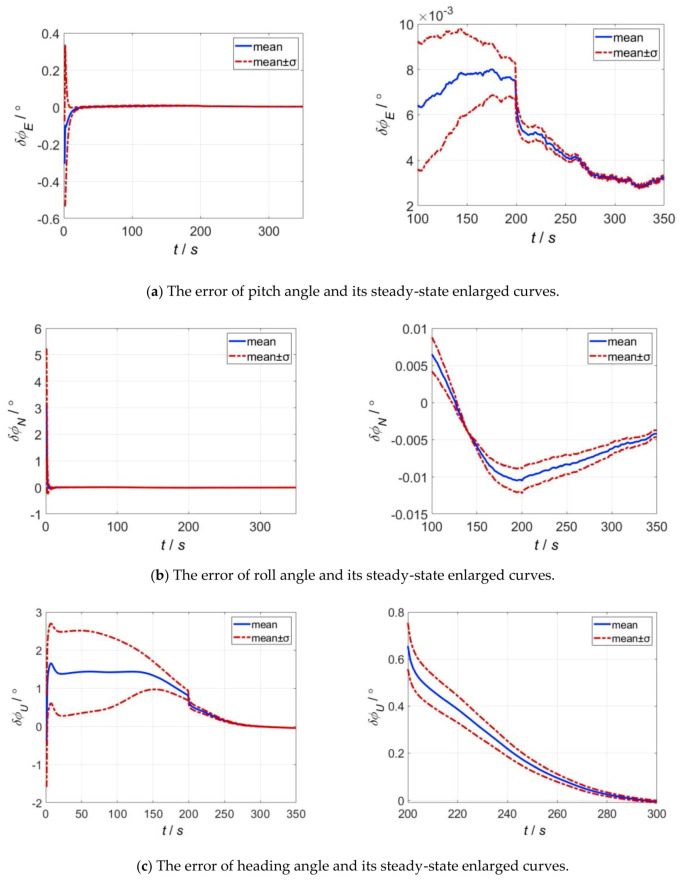
Statistical curves of alignment errors. (**a**) The error of pitch angle; (**b**) the error of roll angle; (**c**) the error of heading angle.

Simulation 1: in order to evaluate the alignment accuracy of the designed nonlinear alignment algorithm with various initial misalignment angles, Monte Carlo simulation was required. In the simulation, the initial values of misalignment angles are set as follows: both the pitch and roll misalignment angles follow the uniform distribution on the interval from −40° to 40°, i.e., $\phi_{E}∼U\left(-40{}^{\circ},40{}^{\circ}\right)$ and $\phi_{N}∼U\left(-40{}^{\circ},40{}^{\circ}\right)$. The yaw misalignment angle follows the uniform distribution on the interval from −50° to 50°, that is, $\phi_{U}∼U\left(-50{}^{\circ},50{}^{\circ}\right)$. The simulation number of times is 20, and the length of single run time is 350 s. The Monte Carlo simulation results of AFIS-EKF2 filtering alignment are shown in Figure 4a–c. It can be seen from Figure 4a,b that the pitch misalignment angle and roll misalignment angle can converge quickly after the start of the alignment process, and the Root Mean Square Errors (RMSE) of both pitch and roll errors have converged to 0.01° within 50 s. As shown in Figure 4c, the yaw misalignment angle converges slowly, and it can converge to the steady state in about 300 s, the RMSE of steady-state errors is 0.127°. The statistics of estimation errors of misalignment angles at 350 s are shown in Table 2. The simulation results show that the precision of the designed AFIS-EKF2 alignment algorithm is close to the ultimate precision of the traditional static-base analytical alignment, and can meet the requirements of nonlinear alignment. The expressions of ultimate precision of static-base analytical alignment are shown in Equations (36)–(38).

The residual errors of conventional static-base analytical alignment (i.e., the ultimate precision) are expressed as:

(42) $$\delta \phi_{E0}^{}=\nabla_{N}^{}/g$$

(43) $$\delta \phi_{N0}^{}=-\nabla_{E}^{}/g$$

(44) $$\delta \phi_{U0}^{}=\varepsilon_{E}^{}/\omega_{ie}^{}/\cos\left(L\right)$$

**Figure 5:**
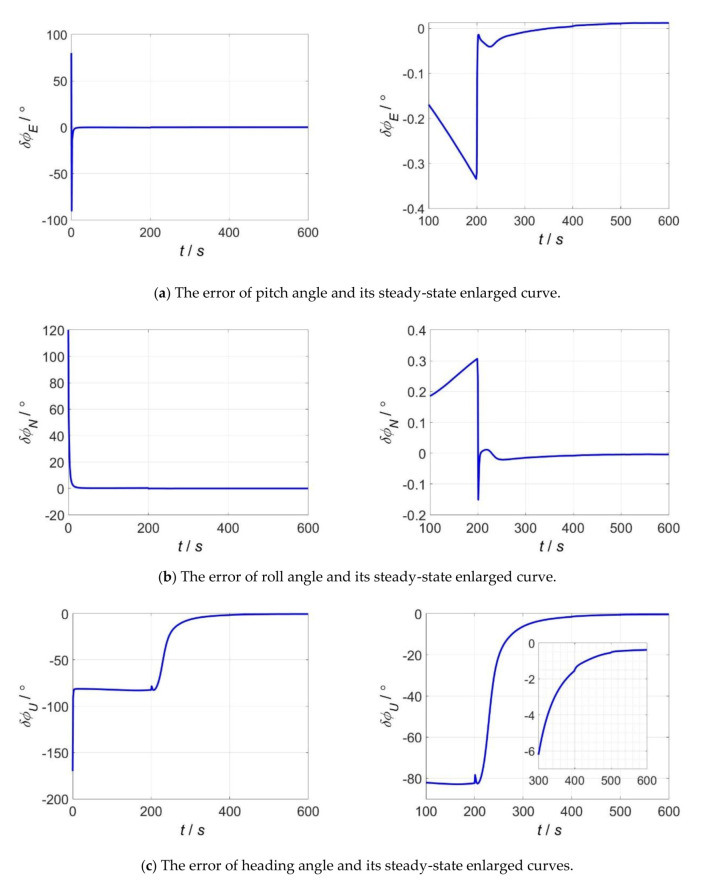
The alignment errors under the condition that initial attitude errors are all very large. (**a**) The error of pitch angle; (**b**) the error of roll angle; (**c**) the error of heading angle.

Simulation 2: In order to verify the accuracy of proposed alignment algorithm under the condition that the initial values of misalignment angles are all very large, the initial values of misalignment angles are set to 80°, 120°, −170°; and the time of fine alignment is 600 s. The simulation results are shown in Figure 5a–c.

In Figure 5a,b, the pitch, roll misalignment angles converge quickly during the process of fine alignment with very large initial values of misalignment angles. Within 50 s, the pitch misalignment angle has converged to −0.1225° and the roll misalignment angle has converges to 0.21°; when the alignment time is 600 s, the estimated residual errors of misalignment angles in pitch and roll are 0.0121° and −0.004°, respectively. It can be seen from Figure 5c that, under the condition that the initial values of three misalignment angles are all very large, the estimate of yaw misalignment angle jumps to the wrong value at the beginning of filtering alignment, and does not converge during the time period from 0 s to 200 s. This is because: In this case, it is difficult to separate and identify the yaw misalignment angle from the error quaternion, the corresponding component of $P_{k}^{}$ converges too early and will be stabilized at the error value. By enabling FIS at 200 s, $P_{k}^{}$ is adaptively adjusted, so that the yaw misalignment angle converges quickly. Finally, the alignment accuracy in yaw reaches −0.3794°.

### 5.2. The Simulation of Fine Alignment on Swaying Base

In order to verify the effectiveness of the proposed nonlinear alignment algorithm under the conditions of large misalignment angles and external angular disturbance, the simulation of filtering alignment on swaying base was performed in this subsection.

#### 5.2.1. Simulation Conditions

The setting of IMU errors is the same as that of previous subsection. The amplitudes of angular disturbances in pitch, roll and yaw are 4°, 6° and 4°, respectively. Moreover, the swaying frequencies in the three directions are all 0.1Hz. The initial values of the pitch, roll and yaw misalignment angles are set to 40°, −40° and 50°, respectively. The velocity measurement noise of nonlinear filtering is assumed to be Gaussian white noise with amplitude of 0.1 m/s. The filtering period is 1 s.

#### 5.2.2. Simulation Results

**Figure 6:**
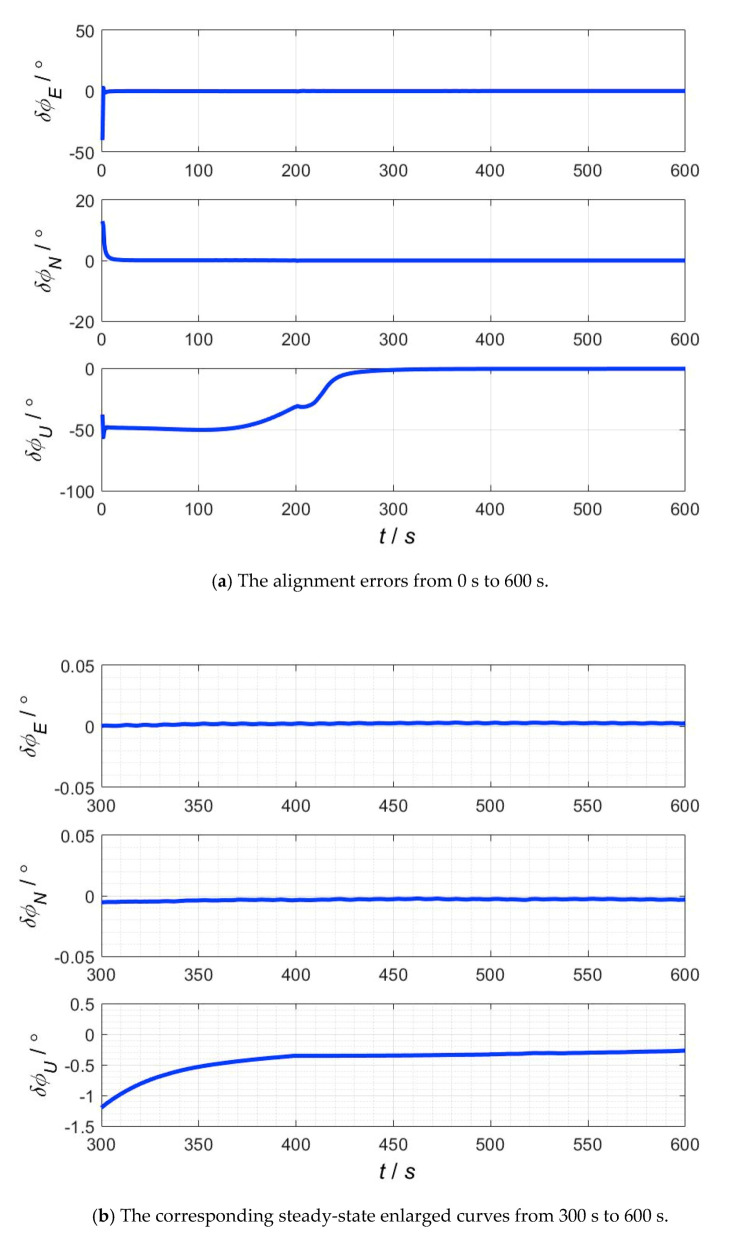
The alignment errors on swaying base and steady-state enlarged curves. (**a**) The alignment errors from 0 s to 600 s; (**b**) the corresponding steady-state enlarged curves from 300 s to 600 s.

The simulation results of the proposed nonlinear alignment on swaying base are shown in Figure 6. The error curves of the pitch and roll converge quickly, and the yaw misalignment angle converges in around 300 s. According to the steady-state data of attitude errors from 300 s to 600 s, the RMSE errors of pitch, roll and yaw are 0.0023°, −0.0032° and −0.2654°, respectively. From the simulation results of fine alignment on swaying base, it can be seen that the heading angle error is −0.26° in 600 s, so the difference between the accuracy of heading alignment and corresponding limit accuracy is a little large. That is because the calculation formulas of limit accuracy used in this paper are derived under ideal conditions of stationary base and only considering the gyro drifts. However, under the conditions of swaying base and large misalignment angles, the observable degree of heading misalignment angle is further decreased due to the influence of model error, disturbance and so on, and the accuracy of heading alignment cannot converge to corresponding limit accuracy. According to the engineering experience and existing research literature, the following conclusion is supported: in nonlinear filtering alignment, the larger the initial misalignment angle or disturbance is, the slower the convergence rate is, and the larger the steady-state error is. The simulation results show that the proposed algorithm is effective under the conditions of large misalignment angles and swaying base.

### 5.3. Experiment on Three-Axis Turntable

In order to further verify the effectiveness of the proposed AFIS-EKF2 algorithm in this paper, the SINS nonlinear filtering fine alignment experiments under different experimental conditions of large misalignment angles were conducted by using FSINS4X fiber-optic-gyroscope (FOG) strapdown inertial navigation system (FSINS4X FOG SINS) on SGT-3T high-precision three-axis turntable.

#### 5.3.1. Experiment Conditions

**Figure 7:**
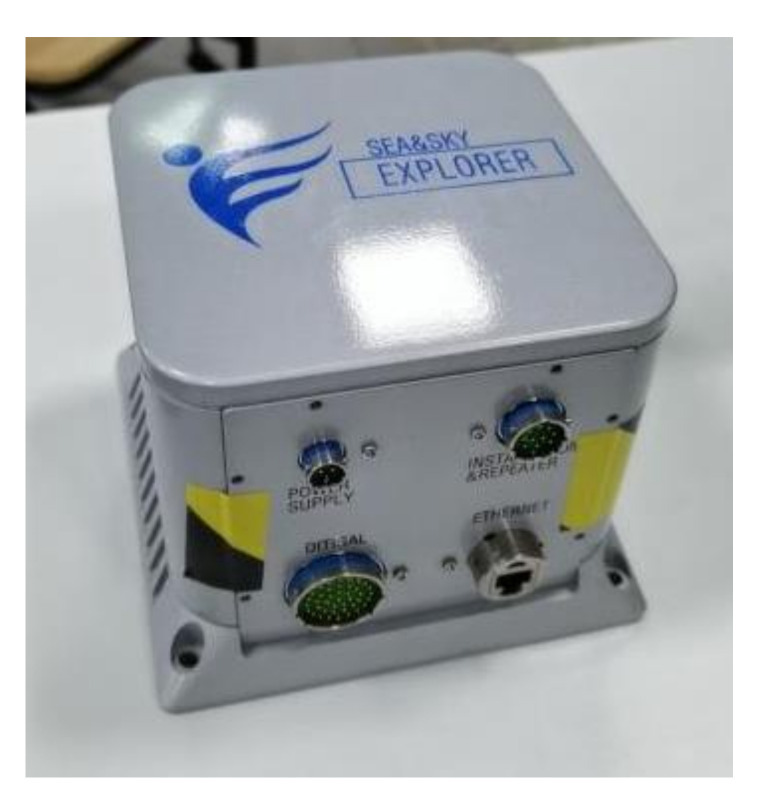
FSINS4X fiber-optic-gyroscope (FOG) strapdown inertial navigation (SINS).

**Figure 8:**
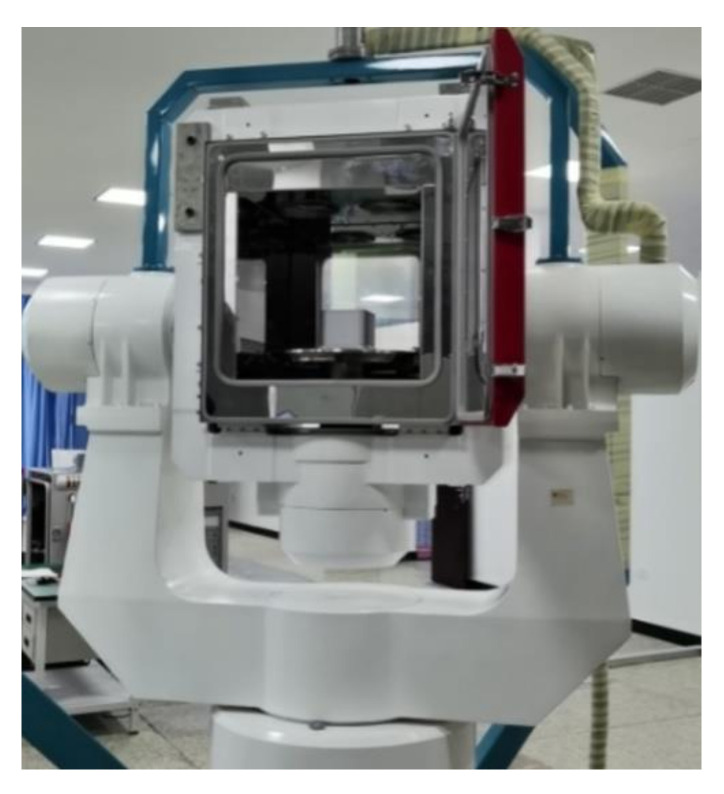
FOG IMU on three-axis turntable.

FSINS4X FOG SINS is composed of three FOG gyroscopes, three quartz flexible accelerometers, navigation computer and power module, etc. FSINS4X FOG SINS is shown in Figure 7, and the main performance parameters of FOG gyroscope and quartz accelerometer are shown in Table 3. The main performance indexes of SGT-3T three-axis turntable are shown in Table 4. FOG SINS is installed on the three-axis turntable through the transition plate, as shown in Figure 8.

In this paper, two groups of experiments were designed to verify the effectiveness of the algorithm, of which Experiment 1 is the alignment experiment under general large misalignment angles, and Experiment 2 is the alignment experiment under extremely large misalignment angles. The ranges of three initial misalignment angles in Experiment 1 are $\phi_{E}\in \left[-40{}^{\circ},40{}^{\circ}\right]$, $\phi_{N}\in \left[-40{}^{\circ},40{}^{\circ}\right]$ $\phi_{U}\in \left[-50{}^{\circ},50{}^{\circ}\right]$, respectively. In Experiment 2, the initial values of three extremely large misalignment angles are set as 80°, 120°, −170°, respectively. The experimental conditions are shown in Table 5.

In the turntable experiment, the position of three-axis turntable in laboratory is (34.2°N, 108.9°E), and various initial large misalignment angles can be set by three-axis turntable. Combined with the preset initial attitude value of SINS, the initial misalignment angle can be set as any large value by adjusting the real attitude of SINS on three-axis turntable at the beginning of alignment. For example, supposing that the initial attitude of SINS is roughly set to be [0, 0, 95]°. In order to set the initial misalignment angle of fine alignment as [35.6, 37.2, 44]°, the real attitude of SINS should be adjusted to (35.6, 37.2, 139.0)° by controlling the rotation of turntable. Before the experiment, the calibration and compensation of SINS were carried out, and the sampling rate of IMU was set as 200 Hz. The three-axis turntable installed with FOG SINS was controlled to return to zero position, and then control the turntable to rotate to the angular position corresponding to the preset large misalignment angles. After the FOG-SINS is powered on and preheated, the fine alignment experiments under large misalignment angles is carried out, and the proposed AFIS-EKF2 algorithm in this paper is verified. In order to analyze the advantages and superiority of the proposed algorithms, several nonlinear alignment methods based on EKF, UKF, PF, FOS, and artificial neural networks (ANN) proposed by other literatures are used for the fine alignment with large misalignment angles under the same experimental conditions. In Experiment 1 and Experiment 2, 20 groups of turntable experiments in different attitudes are carried out for each algorithm of AFIS-EKF2, EKF, UKF, PF, FOS and ANN, respectively. Moreover, the fine alignment time of each experiment is 600 s.

#### 5.3.2. Experiment Results

**Figure 9:**
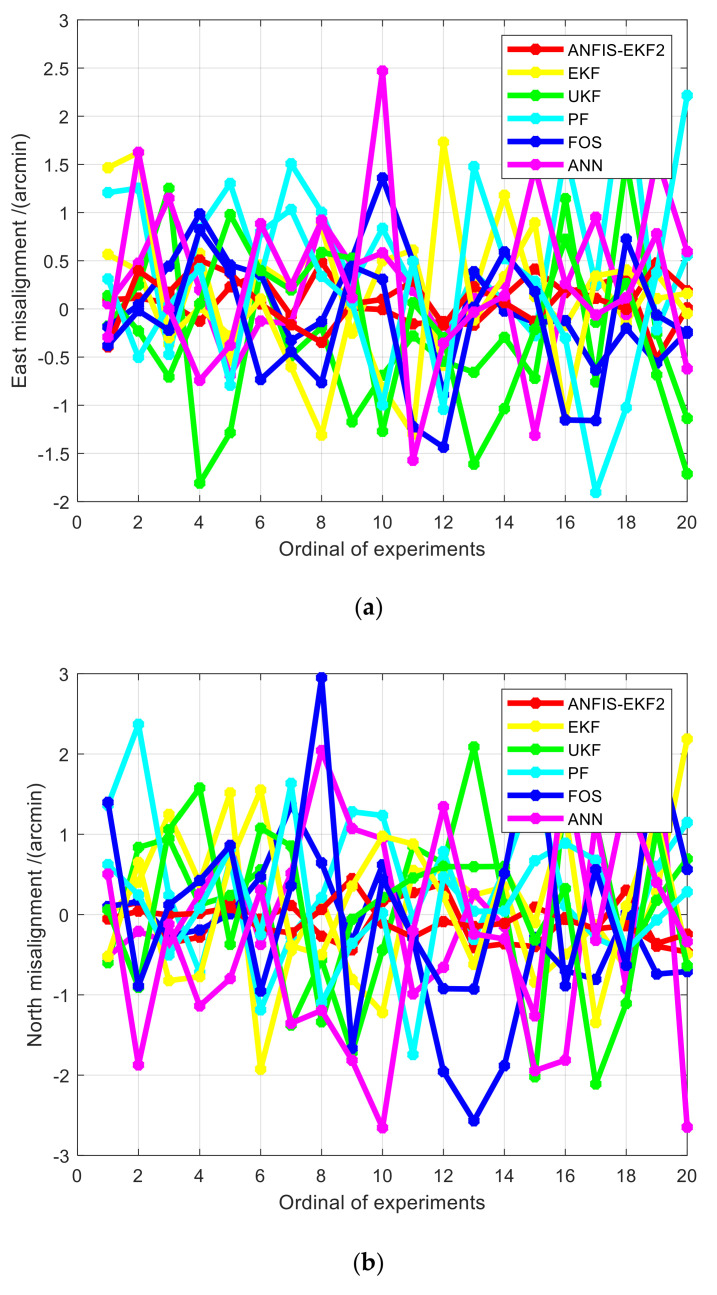

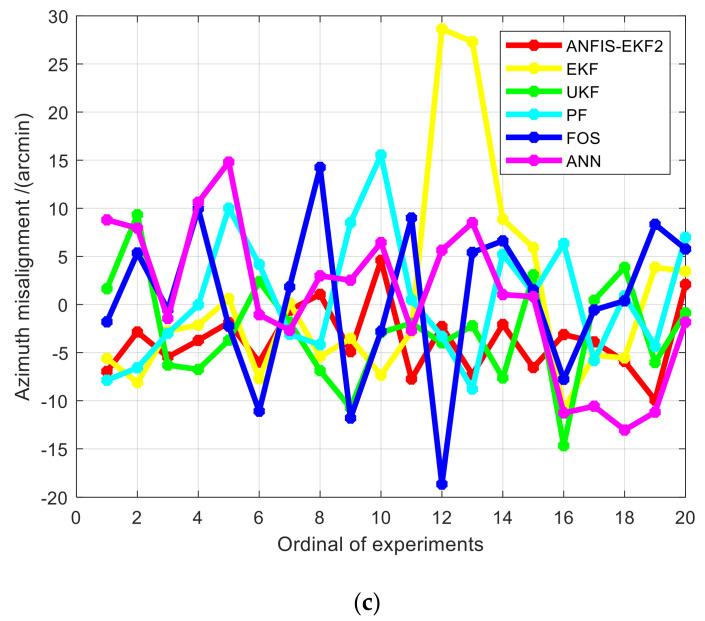
The statistical chart of alignment results of each filtering algorithm in Experiment 1. (**a**) East misalignment; (**b**) north misalignment; (**c**) azimuth misalignment.

In Experiment 1 and Experiment 2, the 20 groups of misalignment angle errors of nonlinear alignment methods based on AFIS-EKF2, EKF, UKF, PF, FOS, and ANN at alignment completion time are counted, and the statistical curves of misalignment angle errors of each filtering algorithm in Experiment 1 are drawn, as shown in Figure 9. In Experiment 2, only the AFIS–EKF2 algorithm proposed in this paper can achieve alignment successfully, and the other five methods are all failed to align, so the statistical data graphs of Experiment 2 are no longer drawn. The Root Mean Square Error (RMSE) of misalignment angle errors of each algorithm in Experiment 1 and Experiment 2 are calculated, as shown in Table 6.

According to the statistical results in Figure 9 and Table 6, the proposed AFIS-EKF2 has higher alignment accuracy, smaller fluctuation and better stability than the other five methods in alignment experiments with large misalignment angles. Moreover, in Experiment 2, only the proposed AFIS-EKF2 method in this paper can successfully complete the fine alignment under extremely large misalignment angles, while the other reference methods have failed in misalignment.

### 5.4. Navigation Experimental Test of SINS on Vehicle

**Figure 10:**
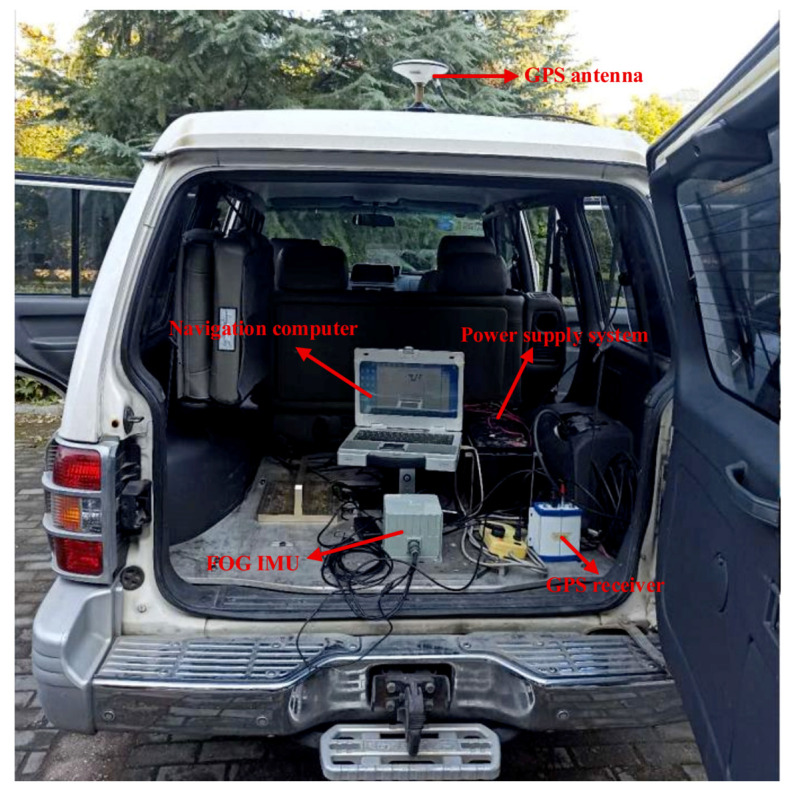
Setup of the experimental platform.

In order to sufficiently check the quality of proposed INS initial alignment algorithms, the navigation test experiment of SINS on a land vehicle (manufactured by Hunan Leopaard Motors Co. Ltd., Changsha, China) was carried out. The experimental platform is shown in Figure 10, which is composed of the Inertial Measurement Unit (FOG-IMU, manufactured by Harbin Engineering University, Harbin, China), global positioning system (GPS) (manufactured by Beijing BDStar Navigation Co., Ltd., Beijing, China), navigation computer (manufactured by EVAK Technology Co., Ltd., Shenzhen, China) and power supply system. Here, a SINS/GPS integrated navigation system is used to provide the reference position and velocity with high accuracy for analyzing the position and velocity errors of pure SINS navigation. The specifications of IMU are: gyro bias stability 0.01°/h, gyro angle random walk $0.001{}^{\circ}/\sqrt{h}$; accelerometer bias stability 50 µg, accelerometer velocity random walk $0.5\;µg/\sqrt{Hz}$ Moreover, the accuracy specifications of GPS are: longitude and latitude errors 1.5 m, altitude error 2.5 m; velocity error 0.03 m/s.

**Figure 11:**
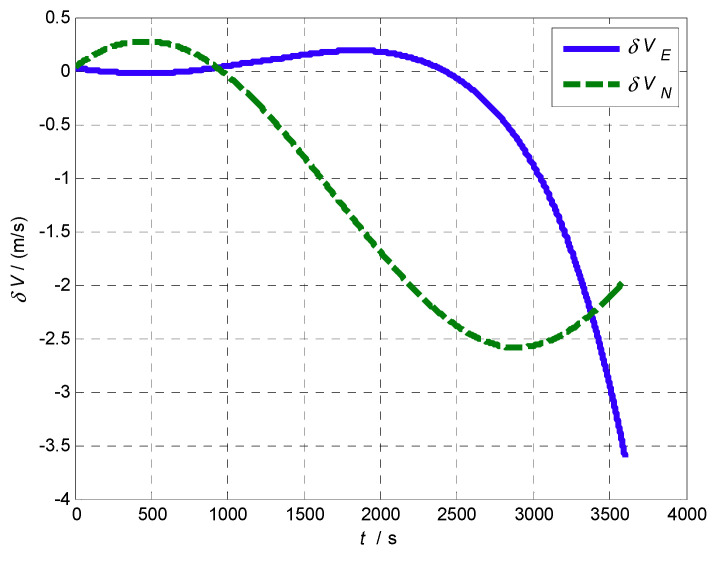
Velocity errors of SINS on a vehicle.

**Figure 12:**
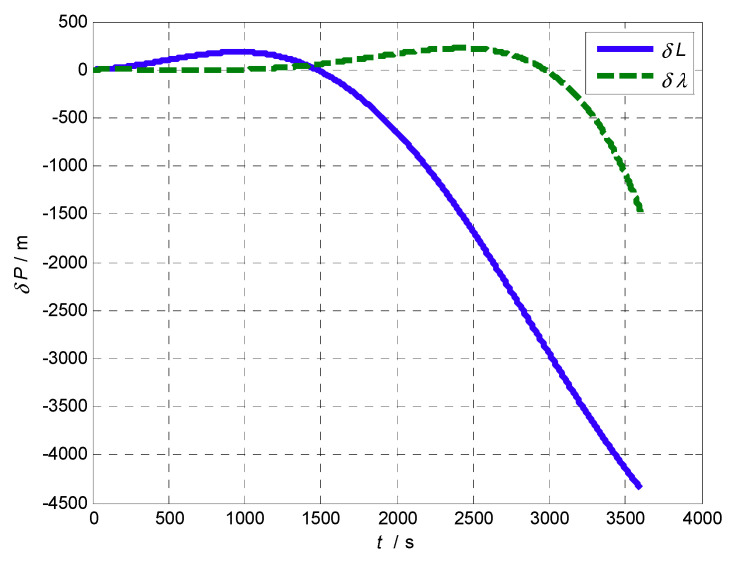
Position errors of SINS on a vehicle.

The experimental steps are as follows: Firstly, the proposed AFIS-EKF2 alignment method is performed for SINS initial alignment with large misalignment angles under the condition of vehicle idling; after initial alignment, INS switches to the inertial mode, the SINS navigation is carried out for a one hour duration; finally, the levels of SINS errors are analyzed by compared with reference velocity and position from SINS/GPS integrated navigation system. The velocity errors of SINS are shown in Figure 11 and position errors of SINS are shown in Figure 12.

It can be seen from Figure 11 and Figure 12 that the velocity errors and position errors of SINS have the trend of slow oscillation and divergence in the navigation time of 1 h, which is consistent with the error propagation characteristics of SINS in theory. The velocity errors and position errors at some time points (500 s, 1000 s, 1500 s, 2000 s, 2500 s, 3000 s, 3500 s, 3600 s) are listed in Table 7.

## 6. Conclusions

Under the conditions of unknown initial attitudes or strong disturbance, the error equations of SINS have strong nonlinearity. In such conditions, the linear error models of SINS are inconsistent with the reality. The filtering alignment based on classical linear KF has the disadvantages of too long convergence time or filtering divergence. In this paper, a novel SINS fine alignment method which is based on AFIS-EKF2 filter is proposed to realize the fine alignment of SINS with large misalignment angles. The simulation analyses were performed, which include the Monte Carlo simulation on stationary base, alignment simulation with very large misalignment angles and alignment simulation on swaying base. The simulation results show that the AFIS-EKF2 filtering algorithm is suitable for SINS fine alignment with arbitrarily large misalignment angles. The turntable experiment was performed, and the effectiveness and superiority of the proposed method were further verified by compared with other nonlinear methods. The proposed fine alignment algorithm has important practical significance for some important special application scenarios of too short coarse alignment time (e.g., the missile launch vehicle under the threat of artillery attacks) or the fine alignment without coarse alignment process (e.g., missile-borne SINS).

## Figures and Tables

**Table 1:** Fuzzy subsets of input variable $c_{k}^{}$.

Fuzzy Subsets	Domain	Membership Functions	Break Points
Small (S)	[0, 3]	zmf	[0.8, 1.2]
Large (L)	[0, 3]	smf	[0.8, 1.2]

**Table 2:** Statistical properties of alignment errors.

Errors (°)	Pitch Errorδθ	Roll Errorδγ	Yaw Errorδψ
RMSE	0.0032	0.0042	0.1271
Limits	0.0029	−0.0029	0.0924

**Table 3:** Main parameters of gyro and accelerometer.

Parameters	Value
Gyro measurement range	±600°/s
Gyro bias stability	<0.01°/h
Gyro angle random walk	<0.001°/h^1/2^
Accelerometer measurement range	±20 g
Accelerometer bias stability	<50 µg
Accelerometer velocity random walk	<5 µg/h^1/2^

**Table 4:** Main indexes of SGT-3T turntable.

Parameters	Value
Gyration accuracy	±2″
Rotation range	0~360°
Angle accuracy	±2″
Angular repeatability	±1″
Angular rate range	0.001~200°/s
Angular rate accuracy	5 × 10^−4^°/s

**Table 5:** Settings of experiment condition.

Experiments	Initial Misalignment Angles		
	East	North	Azimuth
Experiment 1	$\phi_{E}\in \left[-40{}^{\circ},40{}^{\circ}\right]$	$\phi_{N}\in \left[-40{}^{\circ},40{}^{\circ}\right]$	$\phi_{U}\in \left[-50{}^{\circ},50{}^{\circ}\right]$
Experiment 2	80°	120°	−170°

**Table 6:** Comparison of alignment experiment results of AFIS–EKF2, EKF, unscented Kalman filter (UKF), particle filter (PF), and fast orthogonal search (FOS).

Experiments	Method	Misalignment Angle Error RMSE (′)		
		East	North	Azimuth
Experiment 1	AFIS-EKF2	0.2280	0.2520	4.5720
	EKF	0.7079	0.9360	8.1605
	UKF	0.8164	0.9245	6.5188
	PF	0.8980	0.8782	5.8669
	EKF-based FOS	0.7787	1.0828	9.4473
	EKF-based ANN	0.9730	1.1793	8.5884
Experiment 2	AFIS-EKF2	0.7302	−0.2396	−22.7640
	EKF	Failure		
	UKF	Failure		
	PF	Failure		
	EKF-based FOS	Failure		
	EKF-based ANN	Failure		

**Table 7:** Errors of SINS on a vehicle.

*t* (s)	δ*V_E_* (m/s)	δ*V_N_* (m/s)	δ*L* (m)	δ*λ* (m)
500 s	−0.018818	0.27268	101.89	−1.3953
1000 s	0.045686	−0.044207	183.29	0.72195
1500 s	0.14902	−0.80396	−16.265	59.255
2000 s	0.17986	−1.6915	−644.07	167.41
2500 s	−0.065639	−2.3809	−1680.2	220.19
3000 s	−0.88205	−2.5697	−2946.9	−24.945
3500 s	−2.9383	−2.1155	−4142.2	−1083.3
3600 s	−3.6086	−1.9593	−4345.4	−1474.5
